# Quality of life data as prognostic indicators of survival in cancer patients: an overview of the literature from 1982 to 2008

**DOI:** 10.1186/1477-7525-7-102

**Published:** 2009-12-23

**Authors:** Ali Montazeri

**Affiliations:** 1 Iranian Institute for Health Sciences Research, ACECR, Tehran, Iran; 2 Public Health and Health Policy, Division of Community Based Sciences, University of Glasgow, Glasgow, UK

## Abstract

**Background:**

Health-related quality of life and survival are two important outcome measures in cancer research and practice. The aim of this paper is to examine the relationship between quality of life data and survival time in cancer patients.

**Methods:**

A review was undertaken of all the full publications in the English language biomedical journals between 1982 and 2008. The search was limited to cancer, and included the combination of keywords 'quality of life', 'patient reported-outcomes' 'prognostic', 'predictor', 'predictive' and 'survival' that appeared in the titles of the publications. In addition, each study was examined to ensure that it used multivariate analysis. Purely psychological studies were excluded. A manual search was also performed to include additional papers of potential interest.

**Results:**

A total of 451 citations were identified in this rapid and systematic review of the literature. Of these, 104 citations on the relationship between quality of life and survival were found to be relevant and were further examined. The findings are summarized under different headings: heterogeneous samples of cancer patients, lung cancer, breast cancer, gastro-oesophageal cancers, colorectal cancer, head and neck cancer, melanoma and other cancers. With few exceptions, the findings showed that quality of life data or some aspects of quality of life measures were significant independent predictors of survival duration. Global quality of life, functioning domains and symptom scores - such as appetite loss, fatigue and pain - were the most important indicators, individually or in combination, for predicting survival times in cancer patients after adjusting for one or more demographic and known clinical prognostic factors.

**Conclusion:**

This review provides evidence for a positive relationship between quality of life data or some quality of life measures and the survival duration of cancer patients. Pre-treatment (baseline) quality of life data appeared to provide the most reliable information for helping clinicians to establish prognostic criteria for treating their cancer patients. It is recommended that future studies should use valid instruments, apply sound methodological approaches and adequate multivariate statistical analyses adjusted for socio-demographic characteristics and known clinical prognostic factors with a satisfactory validation strategy. This strategy is likely to yield more accurate and specific quality of life-related prognostic variables for specific cancers.

## Background

Health-related quality of life is now considered an important end-point in studies of outcomes in oncology. Studies of quality of life have several benefits when they show evidence that the measurements were conducted and reported appropriately [1]. One benefit is that information obtained from such studies can indicate the directions needed for more efficient treatment of cancer patients. In addition, it has been shown that quality of life assessments in cancer patients may contribute to improved treatment and could even be of prognostic value [2-7].

However, it is believed that health-related quality of life is only a single type of patient-reported outcome. Patient-reported outcome is an 'umbrella term' encompassing any outcome reported by a patient himself or herself based on perception of a disease and its treatment, such as health-related quality of life, functional well-being and satisfaction [8]. This approach is currently receiving more attention and many believe it could help both physicians and patients, and even family carers to achieve a better understanding of the treatment outcomes of cancer patients and make appropriate decisions.

Using either term - 'patient-reported outcome' or 'health-related quality of life' - the evidence compiled suggests that information provided by cancer patients via quality of life measures is very helpful for clinical decision-making and better patient management. For instance, a recent review on health-related quality of life assessment in leukaemia randomised controlled trials showed how quality of life assessments would have added value in supporting clinical decision-making. The review of 3838 leukaemia patients indicated that 'imatinib' greatly improved health-related quality of life compared to 'interferon-based' treatment in chronic myeloid leukaemia patients. The review concluded that health-related quality of life assessment is feasible in randomised trials and has the great potential of providing valuable outcomes to further support clinical decision-making [9]. As suggested 'the main advantage of this line of research is that of potentially providing clinicians with a more accurate picture of the patient's prognostic profile, hence possibly further improving accuracy of prognosis and making more tailored treatment decisions' [10].

In addition, since lengthening survival of many or most cancer patients is considered paramount in every effort at treatment, the clinical implications of relationship between quality of life data and survival could be regarded as very important. Thus, many investigators from both clinical oncology and health sciences research have begun demonstrating that health-related quality of life in cancer patients could be associated with survival duration. In fact, this group of investigators has sought to justify the collection of quality of life information, even if only to assess improved survival as the main outcome in cancer care. They believed that quality of life data may not only be helpful in evaluating cancer care outcomes from patients' or family carers' perspectives but may also, like clinical information, be prognostic or predictive of survival duration, thus helping clinicians to reach better decisions on patient management or identify their needs and decide on possible additional interventions, such as referral for counselling or psychosocial help and support. Therefore, biomedical journals have for many years been publishing reports that focus on the relationship between quality of life data and survival duration.

The aim of this review was to examine the literature on the relationship between quality of life data and survival duration since the topic first appeared in English biomedical journals. The intention was to compile the evidence so far obtained, contribute to existing knowledge, and help both researchers and clinicians to achieve a better profile on the topic, and consequently aid in improving the quality of life of cancer patients.

## Methods

### Search engines and time period

A literature search was carried out using MEDLINE, EMBASE, the Science Citation Index (ISI), the Cumulative Index to Nursing and Allied Health Literature (CINAHL), the PsycINFO, the Allied and Complementary Medicine (AMED) and Global Health databases to assess the existing knowledge about the relationship between quality of life data as 'prognostic' or 'predictive' indicators and survival in cancer patients. The aim was to review all full publications that appeared in English language biomedical journals between 1982 and 2008. The year 1982 was chosen because the first study on the relationship between survival and quality of life data was published in that year.

### Definitions

- Health-related quality of life was defined as an individual's perceived physical, mental and social health status affected by cancer diagnosis or treatment. This article uses the terms 'health-related quality of life' and 'quality of life' interchangeably.

- Health-related quality of life measures (instruments, questionnaires) were defined as well-established questionnaires that measure individuals' perceptions of their own physical, mental and social health status, or some aspects of their health status resulting from cancer and its treatment.

- Health-related quality of life data were defined as the data collected using valid generic or specific health-related quality of life measures.

- Predictive or prognostic indicators were defined as any independent variables (e.g. health-related quality of life parameters) that can be used to estimate the chance of a given outcome (e.g. survival duration).

### Search strategy

The search strategy was limited to cancer and included the combination of keywords 'quality of life', 'patient-reported outcomes' 'prognostic', 'predictor', 'predictive', and 'survival' in the titles of publications. This provided the initial database for the review. A manual search also was performed to include possible additional papers.

### Inclusion and exclusion criteria

In addition to publication titles, the literature was examined to ensure that the study used a quality of life instrument or measured quality of life using proxy indicators, and applied multivariate analyses for survival adjusted for one or more known clinical prognostic indicators. Purely psychological studies were excluded. These were defined as studies limited to the relationship between one or more psychological variables, such as fighting spirit, cancer personality, coping styles, hostility, etc. and survival duration.

### Data synthesis

Data obtained from each single study were synthesized by providing descriptive tables reporting authors' names, publication year, study sample, type of cancer (where relevant data were available), instrument used to measure quality of life, and the main findings or conclusions. The findings were then sorted and presented chronologically.

## Results

### Statistics

**Figure 1:**
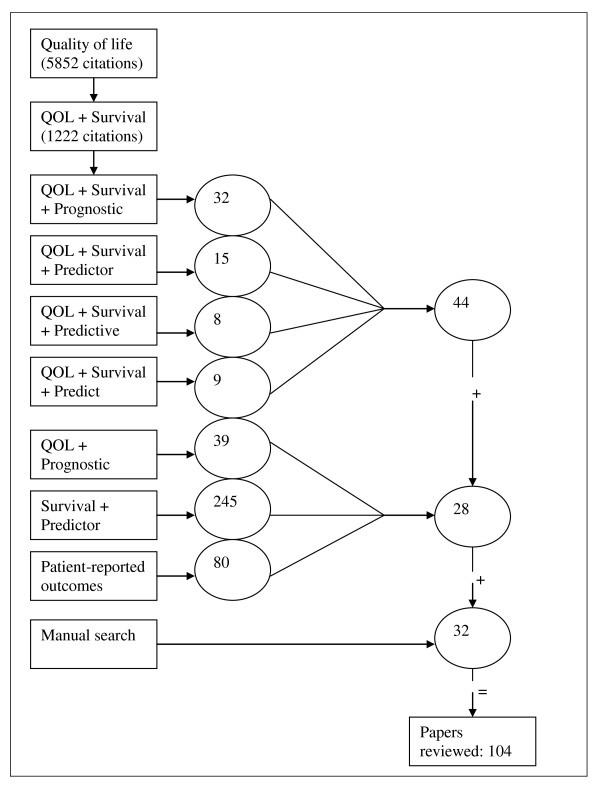
**A schematic picture of the search strategy limited to cancer patients with indicated keywords in titles of publications (numbers are frequency of citations)**.

In total, 451 citations were identified in this systematic review of the literature. After exclusion of duplicates, the abstracts of all citations were reviewed. Of these, 104 citations concerning the relationship between quality of life and survival were found to be relevant and were further examined (Figure 1). Here, the major findings are summarized and presented under the following headings.

### Early pivotal publications [1982-1989]

During the 1980s, a few papers reported positive relationships between some psychosocial and quality of life parameters and survival time in cancer patients. The first paper on this relationship was published in 1982. In that paper the existing records of 651 patients with bronchogenic carcinoma were assessed to determine the relationship between survival and four '*non-anatomical*' prognostic indicators: symptomatic history, performance status, weight loss and age. Adjusting for stage, histological factors and treatment, the analysis showed that weight loss and performance status were significantly associated with survival [11]. In 1985, Cassileth et al. studied 359 cancer patients and found no association between social and psychological factors and duration of survival or time to relapse. They did not collect data on health-related quality of life but concluded that, although these factors may contribute to the initiation of morbidity, the biology of the disease appears to predominate, overriding the potential influence of life-style and psychosocial variables once the disease process has been established [12]. The third paper on the topic appeared in 1987. This paper compared quality of life during chemotherapy for advanced breast cancer between patients receiving intermittent and continuous treatment strategies. The findings indicated that changes in the quality of life index, measured by a series of Linear Analog Self Assessment (LASA) scales for physical well-being, mood, pain and appetite, were independent prognostic indicators of subsequent survival [13]. Kaasa et al. also published a paper on the topic in 1989, in which for inoperable non-small-cell lung cancer they showed that general symptoms and psychological well-being were the best predictors of survival duration [14].

### Heterogeneous sample of cancer patients

Some studies included a heterogeneous sample of cancer populations [15-21]. Global quality of life and physical, social, emotional and cognitive functioning were found to be independent prognostic indicators of survival.

A number of studies showed that global quality of life or global health status was associated with survival time [17-19]. In a study of 253 patients with different cancer diagnoses, Ringdal et al. [16] performed Cox regression analysis adjusted for clinical, demographic and psychosocial factors. They found that physical functioning was an independent predictor of survival time, but psychosocial covariates were not. The results are shown in Table 1.

**Table 1:** Studies on relationship between quality of life data and survival in heterogeneous sample of cancer patients

Author(s)	Year	Sample	HRQOL measure(s)	Results*
Degner and Sloan [15]	1995	435 ambulatory heterogeneous sample of cancer patients (including 82 lung cancer)	SDS	The single measure of symptom distress was a significant predictor of survival in lung cancer.

Ringdal et al. [16]	1996	253 heterogeneous sample of cancer patients	Physical functioning + psychosocial variables	Physical functioning was prognostic factor of survival but psychosocial covariates were not.

Tamburini et al. [17]	1996	100 terminal cancer patients	TIQ	Confusion, cognitive status and global health status were independent prognostic of survival.

Coates et al. [18]	1997	735 advanced malignancies	EORTC QLQ-C30	Global QOL and social functioning were significantly predictive of survival among solid tumor patients, metastatic site.

Dancey et al. [19]	1997	474 heterogeneous population of cancer patients	EORTC QLQ-C30	Global QOL was significantly associated with survival.

Chang et al. [20]	1998	218 cancers patients (colon, breast, ovary or prostate)	MSAS	Physical symptom subscale score significantly predicted survival.

Lam et al. [21]	2007	170 advanced cancer	HDS + ESAS + McGill QOL	ESAS score was independent prognostic factor for survival.

Abbreviations: EORTC QLQ-C30: European Organization for Research and Treatment of Cancer Quality of Life Core Questionnaire; ESAS: Edmonton Symptom Assessment System; HDS: Hamilton Depression Scale; McGill QOL: McGill quality of Life-single item; MSAS: Memorial Symptom Assessment Scale; QOL: quality of life; SDS: Symptom Distress Scale; TIQ: Therapy Impact Questionnaire.

* All results obtained from multivariate analyses after controlling for one or more demographic and known biomedical prognostic factors.

### Lung cancer

Relatively more studies have examined the relationship between quality of life data and survival in lung cancer patients [11,14,22-45]. These studies included either a sample of both small-cell and non-small-cell lung cancer patients, or mostly advanced non-small-cell patients. Two of these 25 studies reported that the overall quality of life score was not a predictor of survival [28,44]. In most instances, baseline overall or global quality of life scores were independent prognostic indicators of survival duration. A clinical trial using FACT-L showed that a higher baseline physical well-being score was not only associated with a better response to treatment (odds ratio = 1.09; P < 0.001) and lower risk of death (risk ratio 0.95; P < 0.001), but also showed that the patient-reported health change during chemotherapy was a significant predictor of clinical outcomes [35]. In contrast, a small-scale study (n = 30, non-small cell lung cancer) using a similar instrument showed no association between the change in quality of life score and survival [31]. In addition, most studies have shown that pain and appetite loss are independent determinants of overall survival. One found that a 40-point increase in the pain subscale of the EORTC QLQ-C30 was associated with a 27% increase in the rate-of-dying hazard [27]. Similarly, Efficace et al. found that a 10-point worsening in the pain and dysphagia scores in a sample of 391 advanced non-small-cell lung cancer patients resulted in a hazard ratio of 1.11 and 1.12, equivalent to 11% and 12% increases in the likelihood of death, respectively [41]. However, psychological distress in lung cancer patients was also associated with survival duration. A study of 133 lung cancer patients using the Self-rating Depression Scale (SDS) indicated that item 19 ("I feel that others would be better off if I were dead") emerged as the most significant predictor of survival duration [26]. Table 2 summarizes the results.

**Table 2:** Studies on relationship between quality of life data and survival in patients with lung cancer

Author(s)	Year	Sample	HRQOL measure(s)	Results*
Pater and Loeb [11]	1982	651 bronchogenic carcinoma	Symptomatic history, performance status, weight loss and age	Weight loss and performance status were significantly affected survival.

Kaasa et al. [14]	1989	102 inoperable non-small-cell, limited disease	Psychological well-being + disease-related symptoms + personal functioning + everyday activity	General symptoms and psychological well-being were the best predictive value for survival.

Ganz et al. [22]	1991	40 advanced metastatic lung cancer	FLI-C	A statistically significant relationship was observed between initial patient-rated QOL and subsequent survival.

Ruckdeschel et al. [23]	1994	438 lung cancer	FLI-C	Total FLI-C score was significant predictor of survival.

Loprinzi et al. [24]	1994	1,115 advanced colorectal or lung cancers	A designed patient-completed questionnaire	Patients' assessment of their own performance status and nutritional factors such as appetite, caloric intake, or overall food intake were prognostic of survival.

Buccheri et al. [25]	1995	128 Lung cancer	TIQ	The self-estimated difficulty at work and doing housework were significant independent prognostic determinants of survival.

Buccheri et al. [26]	1998	133 Lung cancer	SDS	Depression was associated with survival. Diverse SDS subscales were associated with survival.

Herndon et al. [27]	1999	206 advanced non-small-cell lung cancer	EORTC QLQ-C30 + Duke-UNC Social Support Scale	Pain was a significant predictor of survival but overall QOL was not.

Langendijk et al. [28]	2000	198 inoperable non-small-cell lung cancer	EORTC QLQ-C30	Global QOL was a strong prognostic factor of survival.

Burrows et al. [29]	2000	85 recurrent symptomatic malignant pleural effusions	KPS	Only the KPS score (score ≥ 70) at the time of thoracoscopy was predictive of survival. Pleural fluid pH, pleural fluid glucose, and EPC scores were not as reliable as initially reported.

Montazeri et al. [30]	2001	129 lung cancer (small and non-small-cell)	NHP + EORTC QLQ-C30 + EORTC QLQ-LC13	Baseline global QOL was most significant predictor of the length of survival.

Auchter et al. [31]	2001	30 non-small cell lung cancer	FACT-L (TOI)	The change in TOI score was not associated with survival. A trend was noted for shorter survival with the largest negative change in TOI score.

Moinpour et al. [32]	2002	222 advanced non-small-cell	FACT-L	Total FACT-L score was predictor of survival.

Nakahara et al. [33]	2002	179 advanced small- and non-small cell lung cancer	Tokyo University Egogram (measure for mental state)	Mental state was prognostic of survival.

Naughton et al. [34]	2002	70 small-cell lung cancer	EORTC QLQ-C30 + CES-D + MOS Social Support Questionnaire + a sleep quality scale	Higher depressive symptoms were borderline significant in predicting decreased survival.

Eton et al. [35]	2003	573 advanced non-small-cell lung cancer	FACT-L + TOI	Baseline physical well-being and TOI scores predicted either survival duration or disease progression respectively.

Dharma-Wardene et al. [36]	2004	44 advanced lung cancer	FACT-G	Baseline FACT-G total score was significantly associated with survival.

Nowak et al. [37]	2004	53 pleural mesothelomia	EORTC QLQ-C30 + EORTC QLQ-LC13	Functional domains and symptom scales (fatigue and pain) demonstrated predictive validity for survival.

Maione et al. [38]	2005	566 advanced non-small-cell lung cancer	ADL + IADL + EORTC QOL-C30 (global QOL)	Baseline global QOL and IADL were significant prognostic factors for overall survival.

Brown et al. [39]	2005	273 non-small-cell lung cancer	EORTC QLQ-C30 + EORTC QLQ-LC17 + DDC	Global QOL, role functioning, fatigue, appetite loss and constipation were prognostic indicators of survival.

Martins et al. [40]	2005	41 locally advanced or metastatic lung cancer	LCSS	Patients' scores on the LCSS appetite and fatigue subscales were independent predictors of survival.

Efficace et al. [41]	2006	391 advanced non-small-cell lung cancer	EORTC QLQ-C30 + EORTC QLQ-LC13	Pain, and dysphagia were significant prognostic factors for survival.

Sundstrom et al. [42]	2006	301 stag III non-small-cell lung cancer	EORTC QLQ-C30	Appetite loss was the most significant prognostic factor of survival.

Bottomley et al. [43]	2007	250 malignant pleural mesothelioma	EORTC QLQ-C30 + EORTC QLQ-LC13	Pain, and appetite loss were independent prognostic indicators of survival.

Fielding and Wong [44]	2007	534 liver and lung cancers	FACT-G	Global QOL scores did not predict survival in liver and lung cancer. Physical well-being and appetite predicted survival in lung cancer.

Jacot et al. [45]	2008	301 non-small-cell lung cancer	LCSS	Pretreatment LCSS global symptoms score was independent determinant of overall survival.

Abbreviations: CES-D: Centre for Epidemiologic Studies-Depression Scale; DDC: Daily Diary Card; EORTC QLQ-C30: European Organization for Research and Treatment of Cancer Quality of Life Core Questionnaire; EORTC QLQ-LC13 (or QLQ LC17): EORTC Lung Cancer specific Quality of Life Questionnaire (previously containing 17items); FACT-G: Functional Assessment of Cancer Therapy-General module; FACT-L: Functional Assessment of Cancer Therapy-Lung module; FLI-C: Functional Living Index-Cancer; IADL: Instrumental Activities of Daily Living; KPS: Karnofsky Performance Status; LCSS: Lung Cancer Symptoms Scale; MOS: Medical Outcomes Study; ADL: Activities of Daily Living; NHP: Nottingham Health Profile; QOL: quality of life; SDS: Self-rating Depression Scale; TIQ: Therapy Impact Questionnaire; TOI: Trial Outcome Index.

* All results obtained from multivariate analyses after controlling for one or more demographic and known biomedical prognostic factors.

### Breast cancer

Studies that examined the relationship between quality of life data and survival in breast cancer patients are presented in Table 3[13,46-63]. Some showed that baseline quality of life predicts survival in advanced breast cancer, but not in early stages of disease [51]. Two recently published papers also confirmed that baseline quality of life was not a prognostic indicator in non-metastatic breast cancer patients. One of these, using Cox survival analysis, indicated that neither health-related quality of life nor psychological status at diagnosis or one year later was associated with medical outcome in women with early-stage breast cancer [59]. The other, on a sample of 448 locally advanced (non-metastatic) breast cancer patients, showed that baseline health-related quality of life parameters had no prognostic value [57]. The latter study reported that the final multivariate model retained inflammatory breast cancer as the only factor predicting overall survival, with a hazard ratio of 1.37 (95% CI = 1.02-1.84). However, a study using the Daily Diary Card to measure quality of life in advanced breast cancer showed that the instrument afforded accurate prognosis of the subsequent response to treatment and survival duration [47]. Similarly, Seidman et al. [48] evaluated quality of life in two phase-II clinical trials for metastatic breast cancer and found that the baseline scores of two validated quality of life instruments independently predicted overall survival. In addition, some studies have demonstrated that certain aspects of quality of life data, including physical health [46], pain [52,55] and loss of appetite [58], were significant prognostic indicators of survival in women with advanced breast cancer. One study also demonstrated that baseline physical aspects of quality of life and its changes were related to survival, but psychological and social aspects were not [53].

**Table 3:** Studies on relationship between quality of life data and survival in patients with breast cancer

Author(s)	Year	Sample	HRQOL measure(s)*	Results*
Coates et al. [13]	1987	226 advanced breast cancer	LASA scores for physical well-being + mood, pain, and appetite (as QOL index)	Changes in QOL scores were independent prognostic of survival.

Coates et al. [46]	1992	226 advanced breast cancer	LASA scores for physical well-being + mood, nausea, vomiting, and appetite (as QOL index)	Both QOL index and physical well-being were independent prognostic factors of survival.

Fraser et al. [47]	1993	60 advanced breast cancer	DDC + LASA + NHP	The DDC provided accurate prognostic data regarding subsequent response and survival.

Seidman et al. [48]	1995	40 advanced breast cancer	MSAS + MSAS-GDI + FLI-C + RMHI + BPI + MPAC	Baseline global QOL and distress index scores independently predicted the overall survival.

Tross et al. [49]	1996	280 early stage breast cancer	SCL-90-R	No significant predictive effect of the level of depression on length of disease-free and overall survival observed.

Watson et al. [50]	1999	578 early stage breast cancer	MAC + CECS + HADS	Depression score of the HADS and helplessness and hopelessness category of the MAC had determinant effect on survival.

Coats et al. [51]	2000	227 metastatic and early stage breast cancer	Physical well-being + mood, appetite, and coping (as QOL index)	Disease-free survival was not significantly predicted by QOL scores at baseline or by changes in QOL scores. After relapse QOL scores were predictive for subsequent survival.

Kramer et al. [52]	2000	187 advanced breast cancer	EORTC QLQ-C30	Pain was prognostic for survival. However, fatigue and emotional functioning were significant in backward selection model.

Shimozuma et al. [53]	2000	47 advanced or end stage breast cancer	QOL-ACD	Physical aspects of QOL were significantly related to survival. The change in scores of both overall QOL and the physical aspects of QOL were also significant predictors of survival.

Butow et al. [54]	2000	99 metastatic breast cancer	Cognitive appraisal of threat + coping + psychological adjustment + perceived aim of treatment + social support + QOL	Minimization was associated with longer survival while a better appetite predicted shorter duration of survival.

Luoma et al. [55]	2003	279 advanced breast cancer	EORTC QLQ-C30	Baseline severe pain was predictive for a shorter overall survival. QOL scores had no great importance in predicting primary clinical endpoints such as time to progression or overall survival.

Winer et al. [56]	2004	474 metastatic breast cancer	FLI-C + SDS	Global QOL and symptom distress scores were prognostic for survival.

Efficace et al. [57]	2004	448 nonmetastatic breast cancer	EORTC QLQ-C30	Baseline QOL had no prognostic value in nonmetastatic breast cancer.

Efficace et al. [58]	2004	275 matastatic breast cancer	EORTC QLQ-C30 + QLQ-BR23	Loss of appetite was a significant prognostic factor for survival.

Goodwin et al. [59]	2004	397 early stage breast cancer	EORTC QLQ-C30 + POMS + PAIS + IES + MACS +ACS + CECS	QOL and psychological status at diagnosis and 1 year later were not associated with medical outcome.

Watson et al. [60]	2005	578 early stage breast cancer	MAC + HADS	Helplessness/hopelessness was a significant predictor of disease-free survival but depression was not.

Lehto et al. [61]	2006	72 localized breast cancer	Coping + emotional expression + perceived support + life stresses + QOL	Longer survival was predicted by a minimizing-related coping while shorter survival was predicted by anti-emotionality, escape coping, and high level of perceived support.

Gupta et al. [62]	2007	251 breast carcinoma	Ferrans and Powers QLI	Baseline patient satisfaction with health and physical functioning and overall HRQOL were significant prognostic of survival.

Groenvold et al. [63]	2007	1588 breast cancer	EORTC QLQ-C30 + HADS	Emotional functioning was predicted overall survival and fatigue was independent predictor of recurrence-free survival.

Abbreviations: ACS: Adjustment to Cancer Scale; BPI: Brief Pain Inventory; CECS: Courtauld Emotional Control Scale; DDC: Daily Dairy Card; EORTC QLQ-C30: European Organization for Research and Treatment of Cancer Quality of Life Core Questionnaire; FLIC: Functional Living Index-Cancer; HADS: Hospital Anxiety and Depression Scale; IES: Impact of Events Scale; LASA: Linear Analog Self Assessment; MAC: Mental Adjustment to Cancer Scale; MPAC: Memorial Pain Assessment Card; MSAS: Memorial Symptom Assessment Scale; MSAS-GDI: Memorial Symptom Assessment Scale-Global Distress Index; NHP: Nottingham Health Profile; PAIS: Psychological Adjustment to Illness Scale; POMS: Profile of Mood States; QLI: Quality of Life Index; QOL: quality of life; QOL-ACD: Quality of Life Questionnaire for Cancer Patients Treated with Anticancer Drugs; RMHI: Rand Mental Health Inventory; SCL-90-R: Symptom Check List-90 items-Revised; SDS: Symptom Distress Scale.

* All results obtained from multivariate analyses after controlling for one or more demographic and known biomedical prognostic factors.

### Gastro-oesophageal cancers

The findings are summarized in Table 4[64-71]. Studies have shown that physical functioning was an important prognostic indicator for survival in this group of cancer patients. Blazeby et al. [65], using the EORTC core and specific quality of life measures in their study of 89 oesophageal cancer patients, showed that a 10-point increase in the physical functioning score corresponded to a 12% reduction in the likelihood of death at any given time (95% CI = 4-18%). Recent studies using the EORTC QLQ-C30 and QLQ-OES18 found that in addition to physical functioning, symptoms such as fatigue, reflux and appetite loss were also independent predictors of survival duration in patients with either gastric or oesophageal cancers [69,70]. Using the same instrument (EORTC QLQ-C30), a large study of 1080 locally-advanced or metastatic oesophago-gastric cancer patients indicated that the global quality of life during pre-treatment was a predictor of survival duration [67]. However, a study of 185 localized oesophageal cancer patients reported that, although fatigue was a predictor of one-year survival, the global quality of life score was not [71].

**Table 4:** Studies on relationship between quality of life data and survival in patients with gastro-oesophageal cancers

Author(s)	Year	Sample	HRQOL measure(s)	Results*
Blazeby et al. [64]	2000	89 oesophageal cancer	EORTC QLQ-C30 + Dysphagia scale of QLQ-OES24	Physical functioning at baseline was significantly associated with survival.

Blazeby et al. [65]	2001	89 oesophageal cancer	EORTC QLQ-C30 + Dysphagia scale of QLQ-OES24	Physical functioning at baseline was significantly associated with survival. After treatment, improved emotional functioning was significantly related to longer survival.

Fang et al. [66]	2004	110 oesophageal squamous cell cancer	EORTC QLQ-C30	Pretreatment physical functioning was the most significant survival predictor while QOL scores during treatment were not. After treatment dysphagia was the most significant predictor.

Chau et al. [67]	2004	1080 locally advanced or metastatic oesophago-gastric cancer	EORTC QLQ-C30	Pretreatment physical and role functioning and global QOL predicted survival.

Park et al. [68]	2008	164 advanced gastric cancer	EORTC QLQ-C30	Social functioning was significant prognostic factor for survival.

Bergquist et al. [69]	2008	96 advanced oesophageal cancer	EORTC QLQ-C30 + QLQ-OES18	Physical functioning, fatigue and reflux were significant prognostic of survival.

McKernan et al. [70]	2008	152 gastric or oesophageal cancer	EORTC QLQ-C30	Appetite loss was significantly independent predictor of survival.

Healy et al. [71]	2008	185 localized oesophageal cancer	EORTC QLQ-C30	Fatigue score was predictive of 1-year survival but global QOL data were not.

Abbreviations: EORTC QLQ-C30: European Organization for Research and Treatment of Cancer Quality of Life Core Questionnaire; QLQ-OES18 (previously QLQ-OES24): EORTC Oesophageal Cancer specific Quality of Life Questionnaire; QOL: quality of life.

* All results obtained from multivariate analyses after controlling for one or more demographic and known biomedical prognostic factors.

### Colorectal cancer

Social functioning as measured by the EORTC QLQ-C30, or health and physical subscales as measured by the Ferrans and Powers Quality of Life Index, were shown to be prognostic for survival in colorectal cancer patients. One study found that the best model for predicting survival included diarrhoea, eating disorders, restlessness, and ability to work and sleep [72]. The results from four clinical trials of 501 locally advanced and metastatic colorectal cancer patients indicated that one-year survival was 38.3% and 72.5% for patients with global quality of life scores below and above the median, respectively [73]. Another study with a sample of 564 patients with advanced colorectal cancer in 10 countries showed that for every 10-point decrease in social functioning score, as measured by the EORTC QLQ-C30, there was a 6% increase in the likelihood of an earlier death [76]. This study was the first external validation (on an independent dataset of patients) of a previously conducted study indicating that social functioning was an independent prognostic factor of survival [75]. The results are shown in Table 5[24,72-76].

**Table 5:** Studies on relationship between quality of life data and survival in patients with colorectal cancer

Author(s)	Year	Sample	HRQOL measure(s)	Results*
Loprinzi et al. [24]	1994	1115 advanced colorectal or lung	A designed questionnaire	Patients' assessment of their own performance status and nutritional factors such as appetite, caloric intake, or overall food intake were prognostic of survival.

Earlam et al. [72]	1996	50 colorectal with liver metastases	RSCL + HADS + SIP	Diarrhea, eating, restlessness, and ability to work and sleep were predictors of survival.

Maisey et al. [73]	2002	501 locally advanced and metastatic colorectal	EORTC QLQ-C30	Baseline physical, role, social, emotional functioning, global QOL and pain, nausea, dyspnea, and sleep difficulties were strong independent predictors of survival.

Lis et al. [74]	2006	177 colorectal	Ferrans and Powers QLI	Health and physical subscale was predictive of survival.

Efficace et al. [75]	2006	299 metastatic colorectal	EORTC QLQ-C30	Social functioning was a prognostic measure of survival beyond a number of previously known biomedical parameters.

Efficace et al. [76]	2008	564 metastatic colorectal	EORTC QLQ-C30	Social functioning was prognostic factor for survival.

Abbreviations: EORTC QLQ-C30: European Organization for Research and Treatment of Cancer Quality of Life Core Questionnaire; HADS: Hospital and Anxiety Depression Scale; QLI: Quality of Life Index; QOL: quality of life; RSCL: Rotterdam Symptom Checklist; SIP: Sickness Impact Profile.

* All results obtained from multivariate analyses after controlling for one or more demographic and known biomedical prognostic factors.

### Head and neck cancer

Since 1998, several papers [77-84] have examined the relationship between survival and health-related quality of life in head and neck cancer (Table 6). Overall, four out of the eight studies showed no clear relationship between health-related quality of life and survival in head and neck cancer. A study of 208 head and neck cancer patients reported that physical functioning, mood and global quality of life did not predict survival. However, the same study showed that patients with less than optimal cognitive functioning had a relative risk of recurrence of 1.72 (95% CI = 1.01-2.93) and a relative risk of dying of 1.90 (95% CI = 1.10-3.26) [78]. The authors speculated that the influence of cognitive functioning on survival in these patients might be related to the use of alcohol.

**Table 6:** Studies on relationship between quality of life data and survival in patients with head and neck cancer

Author(s)	Year	Sample	HRQOL measure(s)	Results*
De Boer [77]	1998	133 head and neck	Self-reported psychosocial and physical functioning	Patients with higher perceived physical abilities were likely to survive more and less likely to develop a recurrence.

de Graeff et al. [78]	2001	208 head and neck	EORTC QLQ-C30 + QLQ-H&N35 + CES-D	Cognitive functioning was predictor of survival while physical functioning; mood and global QOL were not.

Fang et al. [79]	2004	102 advanced head and neck	EORTC QLQ-C30 + EORTC QLQ-H&N35	Baseline fatigue was predictive of survival while changes in QOL scores during treatment was not.

Mehanna and Morton [80]	2006	200 head and neck	AQLQ + LSS + GHQ	QOL at diagnosis was not significant predictor of survival. One year after diagnosis poor life satisfaction score and pain were significant predictors of survival.

Nordgren et al. [81]	2006	89 head and neck	EORTC QLQ-C30	Physical functioning was significant predictor of survival.

Coyne et al. [82]	2007	1093 locally advanced head and neck cancer	Emotional well-being (FACT-G)	Emotional functioning was not an independent predictor of survival.

Siddiqui et al. [83]	2008	1093 locally advanced head and neck cancer	FACT-H&N	The FACT-H&N score was independently predictive of loco-regional control but not overall survival.

Karvonen-Gutierrez et al. [84]	2008	495 head and neck cancer	SF-36, HNQOL	The SF-36 physical component summary score and three domains of the HNQOL (pain, eating and speech) were associated with survival.

Abbreviations: AQLQ: Auckland Quality of Life Questionnaire; CES-D: Centre for Epidemiologic Studies-Depression Scale; EORTC QLQ-C30: European Organization for Research and Treatment of Cancer Quality of Life Core Questionnaire; EORTC QLQ-H&N35: EORTC Head and Neck Cancer specific Quality of Life Questionnaire; FACT-G: Functional Assessment of Cancer Therapy-General module; FACT-H&N: Functional Assessment of Cancer Therapy-Head & Neck module; HNQOL: Head and Neck Quality of Life Questionnaire; GHQ: General Health Questionnaire; LSS: Life Satisfaction Score; QOL: quality of life; SF-36: 36-item Short Form Health Survey

* All results obtained from multivariate analyses after controlling for one or more demographic and known biomedical prognostic factors.

In contrast, a recent study of 495 head and neck cancer patients reported that the SF-36 physical component summary score and three domains of the HNQOL (pain, eating and speech) were associated with survival [84]. A study by Fang et al. using the EORTC QLQ-C30 and EORTC QLQ-H&N35 showed that, while changes in quality of life scores in patients with head and neck cancer during radiotherapy were not correlated with survival, baseline fatigue score was a significant predictor of survival. They reported that an increase of 10 points in the baseline fatigue score corresponded to a 17% reduction in the likelihood of survival [79].

Finally, as Mehanna et al. suggested, the relationship between health-related quality of life and survival in head and neck cancer patients is currently neither strong nor proven, although there is some association between selected psychosocial factors and long-term survival [85].

### Melanoma

Early studies showed no significant relationship between survival and social and psychological factors in melanoma patients [12,86]. However, a number of subsequent studies indicated a significant correlation between quality of life and survival duration. Coates et al., in a study of 152 patients with metastatic melanoma, found that overall quality of life, mood, and appetite were significant predictors of survival [87]. A study of 140 patients with advanced melanoma [90] found that a score of less than 75 points in overall quality of life and physical distress symptoms, as measured by the Rotterdam Symptom Checklist (RSCL), was associated with hazard ratios of 2.31 (95% CI = 1.09-4-90) and 1.92 (95% CI = 1.10-3.36), respectively. The results are summarized in Table 7[12,86-91].

**Table 7:** Studies on relationship between quality of life data and survival in patients with melanoma

Author(s)	Year	Sample	HRQOL measure(s)	Results*
Cassileth et al. [12,86]	1985 and 1988	359 unresectable cancers or early stage melanoma or breast cancer	Social and psychological factors	Social and psychological factors individually or in combined did not influence the length of survival.

Coates et al. [87]	1993	152 metastatic melanoma	LASA scales + Spitzer QLI	QLI and LASA scores for mood, appetite, and overall QOL were significant predictors of survival.

Butow et al. [88]	1999	125 metastatic melanoma	Cognitive appraisal of threat + coping + psychological adjustment + perceived aim of treatment + social support + QOL	Perceived aim of treatment, minimization, anger and better QOL were independently predictive of longer survival.

Brown et al. [89]	2000	426 early stage melanoma	3 single-item LASA scales measuring physical well-being, mood and perceived effort to cope	Shorter survival duration was associated with a positive mood (On average patients who relapsed or died reported using more active, distraction or avoidant styles of coping).

Chiarion-Sileni et al. [90]	2003	140 advanced melanoma	RSCL	Baseline overall QOL and the physical symptom distress scores were significant independent prognostic factors for survival.

Lehto et al. [91]	2007	59 localized melanoma	Coping with cancer + anger expression + perceived social support + life stresses + domains of QOL	Anger non-expression, hopelessness, over-positive reporting of QOL reduced survival while denial/minimizing response to the diagnosis as such predicted longer survival.

Abbreviations: LASA: Linear Analog Self Assessment; QLI; Quality of Life Index; QOL: quality of life; RSCL: Rotterdam Symptom Checklist

* All results obtained from multivariate analyses after controlling for one or more demographic and known biomedical prognostic factors.

### Other cancers

Studies of the relationship between quality of life data and survival have been reported for brain, ovarian, liver, bladder and other cancer populations. The findings are presented in Table 8[44,92-114]. Except for a few studies of liver, brain and ovarian cancer patients [44,95,112], most found a significant relationship between quality of life scores and survival duration in these patients. A study of 468 patients with multiple myeloma, measuring quality of life by the EORTC QLQ-C30 [94], found that at 12 months follow-up the relative risk of death for a physical functioning score of 0-20 versus a score of 80-100 was 5.63 (99% CI = 2.76-11.49). A study of 233 patients with unresectable hepatocellular carcinoma [103] showed that the hazard ratios for worse appetite score and better physical and role functioning scores, as measured by the EORTC QLQ-C30, were 1.07, 0.91 and 0.94, respectively. However, Mauer et al. in their two studies of brain cancer [107,108] argued that while classical techniques (regression analyses) showed a positive relationship between quality of life data and survival duration, more refined analyses suggested that baseline health-related quality of life scores add relatively little to clinical factors for predicting survival.

**Table 8:** Studies on relationship between quality of life data and survival in patients with other cancers

Author(s)	Year	Sample	HRQOL measure(s)	Results*
Andrykowski et al. [92]	1994	42 leukemia	Depressed mood + Functional QOL + MAC	Anxious preoccupation and functional QOL were independent predictors of survival.

Tannock et al. [93]	1996	161 symptomatic hormone-resistant prostate	EORTC QLQ-C30 + QLQ-PR25 + PROSQOLI	Appetite loss, pain, and physical functioning were associated with survival.

Wisloff and Hjorth [94]	1997	468 multiple myeloma	EORTC QLQ-C30	Physical functioning was independent prognostic factor of survival.

Meyers et al. [95]	2000	80 brain (recurrent glioblastoma multiforme or anaplastic astrocytoma)	FACT-Br + ADL	Measures of QOL and ADL were not independently related to survival.

Jerkeman et al. [96]	2001	95 aggressive lymphoma	EORTC QLQ-C30	Pretreatment global QOL was an independent prognostic marker of overall survival.

Roychowdury et al. [97]	2003	364 locally advanced and metastatic bladder	EORTC QLQ-C30	Longer survival was associated with high physical functioning, low role functioning and no anorexia.

Sehlen et al. [98]	2003	153 brain tumors	FACT-G	The FACT-G sum score was a significant predictor of survival.

Collette et al. [99]	2004	391 symptomatic metastatic hormone-resistant prostate cancer	EORTC QLQ-C30	Insomnia and appetite loss were significant independent predictors of survival.

Monk et al. [100]	2005	179 advanced cancer of cervix	FACT-G + Cervix subscale + FACT/GOG-Ntx+ BPI	Baseline FACT-Cx (FACT-G + Cervix subscale) scores was associated with survival.

Brown et al. [101]	2005	273 brain (high grade gloima)	LASA scales (to measure overall QOL)+ FACT-Br + Fatigue (SDS) + Sleep (ESS) + depression (POMS-SF)+ Mental health (MMSE)	Changes in QOL measures over time were not found to be associated with survival.

Brown et al. [102]	2006	194 brain (high grade glioma)	LASA scales (to measure overall QOL)+ FACT-Br + Fatigue (SDS) + Sleep (ESS) + depression (POMS-SF) + Mental health (MMSE)	Fatigue was significant independent predictor of survival.

Yeo et al. [103]	2006	233 unresectable hepatocellular	EORTC QLQ-C30	Appetite loss, physical and role functioning scores were significant predictor of survival.

Lis et al. [104]	2006	55 pancreatic cancer	Ferrans and Powers QLI	Health and physical subscale was marginally significant predictor of survival.

Dubois et al. [105]	2006	202 refractory multiple myeloma	EORTC QLQ-C30 + QLQ-MY24 + FACIT-F + FACT/GOG-Ntx	Fatigue was significant predictor of survival.

Sullivan et al. [106]	2006	809 metastatic hormon-refractory prostate	EORTC QLQ-C30 + FACT-P	Baseline QOL scores (global QOL, physical, role, and social functioning and pain, fatigue and appetite loss) were significant predictors of survival.

Mauer et al. [107]	2007	247 brain (anaplastic oligodenroglimas)	EORTC QLQ-C30 + EORTC QLQ-BN20	Emotional functioning, communication deficit, future uncertainty, and weakness of legs were significant prognostic of survival. Baseline QOL scores added little to clinical factors to predict survival.

Mauer et al. [108]	2007	490 brain (new diagnosed glioblastoma)	EORTC QLQ-C30 + QLQ-BN20	Cognitive functioning, global health status, and social functioning were significant prognostic factors of survival. Baseline QOL scores added little to clinical factors to predict survival.

Fielding and Wong [44]	2007	358 liver and lung	FACT-G	Global QOL scores did not predict survival in liver and lung cancer. Physical well-being and appetite predicted survival in lung cancer.

Viala et al. [109]	2007	202 multiple myeloma	EORTC QLQ-C30, EORTC QLQ-MY24, FACIT-F, FACT/GOG-Ntx	14 out of 21 patient-reported outcomes were significant predictors of mortality. Clinical plus PRO data increased the predictive power.

Bonnetain et al. [110]	2008	538 advanced hepatocellular carcinoma	Spitzer QLI	Baseline QOL was independent prognostic factor for survival.

Carey et al. [111]	2008	244 advanced ovarian cancer	EORTC QLQ-C30	Performance status and global QOL scores at baseline were prognostic factors for both progression-free survival and overall survival.

Gupta et al. [112]	2008	90 ovarian cancer	Ferrans and Powers QLI	No statistically significant prognostic association of patient satisfaction with QOL was observed with survival.

Robinson et al. [113]	2008	86 pancreatic cancer	FACIT-F+ FAACT + BPI + SF-36	Fatigue strongly predicted survival.

Strasser-Weippl and Ludwig [114]	2008	92 multiple myeloma	EORTC QLQ-C30	Role, emotional, cognitive and social functioning but not physical functioning and global QOL were found to be independent prognostic factors of overall survival.

Abbreviations: ADL: Activities of Daily Living; BPI: Brief Pain Inventory; EORTC QLQ-C30: European Organization for Research and Treatment of Cancer Quality of Life Core Questionnaire; EORTC QLQ-BN20: EORTC Brain Cancer specific Quality of Life Questionnaire; EORTC QLQ-MY24: EORTC Myeloma specific Quality of Life Questionnaire; EORTC QLQ-PR25: EORTC Prostate Cancer specific Quality of Life Questionnaire; ESS: Epworth Sleepiness Scale; FACIT-F: Functional Assessment of Chronic Illness Therapy-Fatigue scale; FACT-Br: Functional Assessment of Cancer Therapy-Brain module; FACT-G: Functional Assessment of Cancer Therapy-General module; FACT-P: Functional Assessment of Chronic Illness Therapy-Prostate module; FAACT: Functional Assessment of Anorexia/Cachexia Therapy; FACT/GOG-Ntx: FACT Gynecologic Oncology Group Neurotoxicity scale; LASA: Linear Analog Self Assessment; MAC: Mental Adjustment to Cancer Scale; MMSE: Folstein Mini-Mental State Examination; POMS-SF: Profile of Mood State-Short Form; PRO: patient-reported outcomes; PROSQOL: Prostate Cancer-Specific Quality-of-Life Instrument; QLI: Quality of Life Index; QOL: quality of life; SDS: Symptom Distress Scale; SF-36: 36-item Short Form Health Survey

* All results obtained from multivariate analyses after controlling for one or more demographic and known biomedical prognostic factors.

## Discussion

Although a helpful review on this topic was published recently [115], the present review, to the author's best knowledge, is the first comprehensive study examining the prognostic value of quality of life data for survival time in cancer patients. The review contained 104 studies and with only a few exceptions, the results in most instances indicated that health-related quality of life data or some quality of life measures were significant predictors of survival duration.

The early studies reported here used *ad hoc *instruments, while more recent studies used well-validated cancer-specific quality of life questionnaires. The most recent studies supplemented their assessments with site-specific questionnaires. Overall, 59 different instruments have been used to measure quality of life in cancer patients [Additional file 1]. The EORTC QLQ-C30 was found to be the most widely used cancer-specific instrument, and as the tables in this review show, the questionnaire often gave fairly consistent and reliable results. In addition, the supplementary EORTC quality of life modules, such as QLQ-BR23, QLQ-LC13 and QLQ-BN20, proved very useful instruments for analysing prognostic indicators, provided that other methodological requisites were ensured. Such instruments could even capture information important to the patients and thus provide better prognostic profiles, enabling clinicians to manage cancer patients more effectively. However, with regard to instruments listed in the tables, one should note that some of them were used for a tailor-specific study, treatment or trial such as the Daily Diary Card (DDC) and the Auckland Quality of Life Questionnaire. Evidently some instruments were well-known generic measures, such as the SF-36, a psychological instrument such the Hospital Anxiety and Depression Scale (HADS), and the General Health questionnaire (GHQ), and/or symptom measures such the Brief Pain Inventory (BPI), and the Symptom Distress Scale (SDS). Therefore the information given in the tables was simply to reflect the variance that existed in the instruments used and neither to convey their psychometric validity nor indicate that they were cancer-specific. As such, the results from studies that used *ad hoc *instruments, a study-specific questionnaire or only general measures should be interpreted with caution.

Many studies reported that the global or the overall quality of life was a significant independent predictor of survival. Global quality of life is a straightforward measure, asking people to evaluate their own health status or quality of life individually (or in combination). It is argued that measures such as global quality of life are patient-rated and thus have the potential to reflect the patient's well-being better than a physician's observed indicators. However, it has (for instance) been recommended that since the global quality of life scale of the EORTC QLQ-C30 is highly correlated with other scales, it should not be included in prognostic indicator analyses when other variables from the EORTC QLQ-C30 are used, in order to achieve model stability [116]. This might explain why a recent review on the association of psychosocial factors with survival in head and neck cancer found that the baseline overall quality of life and depression were not predictors of survival [85]. In addition, when quality of life is included in prognostic indicator analyses, pre-treatment (baseline) and follow-up assessments should be distinguished. Furthermore, the relationship between baseline health-related quality of life data and survival refers to disease-specific characteristics, while follow-up health-related quality of life data and survival in addition refer to treatment-specific characteristics. Indeed baseline data are more often reported to be prognostic because they are more straightforward to assess. However, collecting follow-up data is a major challenge and should be encouraged, since pre-treatment quality of life data were not prognostic for survival times in some cancers, while changes in quality of life scores or follow-up data were usually prognostic in these occasions. More importantly, tumour type and stage of disease are essential for drawing conclusions from such findings. In many studies, quality of life data were prognostic indicators of survival duration in patients with solid tumours and advanced diseases, but not in those with soft tumours and early-stage diseases.

Several measures, such as physical functioning, showed particularly significant associations with survival duration in cancer patients. It is argued that physical functioning might be a surrogate marker for an unrecognized biological prognostic indicator, so a causal association between physical functioning and survival time should not be inferred [65]. In addition, it is argued that since performance status and physical functioning are significantly associated with each other, in many instances when one includes both physical functioning and performance status in the regression models, the likelihood of finding inconsistent results can be expected. In other words, in such circumstances in some studies physical functioning would emerge as an independent prognostic factor and in some others performance status or even in certain cases both might be found prognostic factors for survival duration. Thus, as indicated earlier, the role of physical functioning and performance status in prognostic studies need to be evaluated with caution. A recent meta-analysis of the relationship between baseline quality of life data from the EORTC clinical trials and survival indicated that physical functioning was a significant independent prognostic factor but performance status (as measured by the World Health Organisation performance status) was not [5], whereas a study in metastatic kidney cancer patients reported that both physical functioning and performance status were correlated with a longer progression-free survival [117].

Among symptoms, appetite loss, pain and fatigue at baseline were the most important or strongest independent predictors of survival in many of the studies on different cancer populations. One possible explanation is that these symptoms are very sensitive markers of patient well-being. In addition, as explained by Efficace et al. [58], such findings might arise because quality of life measures in effect mask each other in multivariate analyses, so making variables such as appetite loss or pain or fatigue appear to be the most important or strongest predictors of survival time. Another possible explanation is that such symptoms might reflect, for instance, weight loss, which itself is an important prognostic indicator.

As suggested by Gotay et al. [115], there are several explanations for the association between health-related quality of life data and survival duration in cancer outcome studies. They summarized four possible explanations: (i) quality of life measures include different items and thus provide more sensitive information than traditional performance status and toxicity measures; (ii) quality of life data especially those collected at baseline before disease progression could pick up relevant information earlier than established clinical prognostic factors; (iii) quality of life data are markers of patients' behaviour because they relate to diagnosis, treatment and subsequent outcomes of the disease; and (iv) quality of life data are markers of individual characteristics such as personality style and adapting coping strategies, which affect the disease process and outcomes in cancer patients.

In addition, the relationship between measures such as global quality of life or self-rated health and survival or mortality might be explained in the context of the body-mind relationship [118-120]. For instance, a recent publication on the topic concluded that self-reported health is a unique indicator of human health status; its origins lie in a process whereby information from the individual's body and mind is received, selected, reviewed and summarized and therefore it could predict the most absolute biological events, such as survival or death [121].

The current review, however, suggests an additional explanation that might be helpful in interpreting the findings from studies of the relationship between quality of life data and survival duration. Quality of life data might be markers of the socio-economic status of cancer patients. Evidence for a relationship between socio-economic status and survival time for many cancers is being compiled [e.g. see [122-130]]. In this context, a cancer patient's socio-economic status predicts survival. For instance, cancer patients with higher social class would have a better quality of life [e.g. see [131-134]], and consequently those who report a better quality of life at baseline assessment may live longer. Thus it is not surprising that, in addition to clinical measures, quality of life data are predictive of survival duration. This hypothesis needs further assessment. In future studies on the relationship between quality of life data and survival duration, in addition to biomedical measures, adjustments should be made for patients' socioeconomic status. It would then remains to be seen whether health-related quality of life data still act as significant independent predictors of survival or not. However, the known clinical measures that most studies frequently entered into a multivariate model included age at diagnosis; gender (where necessary); stage (tumour characteristics); occurrence of metastases (or number of metastatic sites involved); weight loss; laboratory parameters (where necessary); performance status and type of treatment. It seems that co-morbidity, and measures of patients' socioeconomic status (for example income, education, occupation, living conditions or social class) are also important to be included in the final model when one considers assessing the relationship between quality of life data and survival duration.

Although this review has included studies that examined the relationship between quality of life data and survival, it excluded purely psychological studies. There are several useful studies on association between psychological data and survival and thus if one wishes to have a better understanding on the topic it is necessary to review these papers as well. For instance, a systematic review of the literature clearly documented the influence of psychological coping on survival and recurrence in cancer patients [135]. The review concluded that there is little consistent evidence that psychological coping style is important in survival from or recurrence of cancer. Similarly, a systematic review of the effect of psychosocial factors on breast cancer outcome indicated that, although most studies on the topic have shown a significant relationship between psychosocial factors and survival, the relevant psychosocial variables were neither consistently measured across studies nor, in many cases, consistent in their findings [136]. In contrast, a recent review on the relationship between stress-related psychosocial factors and survival in cancer patients indicated that stressful life experiences were related to poorer cancer survival and higher mortality. It also suggested that stress-prone personalities or unfavourable coping styles, and negative emotional responses or poor quality of life, were related to poorer cancer survival and higher mortality [137]. However, some papers that belonged in principle to the discipline of psychology were inevitably included in the present review. These papers usually reported that a measure of quality of life had been incorporated in the study, but no well-known instruments were used for the measurements. Contrary to expectation, these papers found that, in multivariate analyses, conditions such as over-positive reporting of quality of life [91] or having a better appetite were indicators of shorter survival [54].

Finally, the inherent limitations and controversial issues related to studies of relationship between survival and quality of life data should not be neglected. For example, many studies reporting on a positive relationship between survival and quality of life data originate from previously conducted randomised clinical trials. Although this is the best-known methodology to evaluate treatments outcomes, it can also be argued that, since patients in randomised clinical trials have highly selected criteria (e.g. no associated co-morbidity), one might wonder whether this association also works in the real world [10]. Perhaps only by testing this hypothesis in an observational setting would it be possible to actually verify whether health-related quality of life parameters have a prognostic value. In addition, since most evidence on positive relationship between quality of life data and survival comes from studies with different patients groups, or studies that used different instruments to measure quality of life, or studies that applied different statistical methodology (and sometimes even inappropriate statistical analysis), thus cross-study comparisons are impossible or very complicated, indicating that current evidence is still inconclusive [138]. With regard to statistical analysis, it is argued that statistical methodology is crucial in prognostic factor analysis of health-related quality of life where different statistical strategies can lead to different findings. Mauer et al. suggest at least two recommendations to increase a substantial accuracy of the prognostic models for relationship between quality of life data and survival: validation strategy, and added prognostic value of health-related quality of life factors analysis. They refer to the former as the only way to avoid over-fitting logistic regression models. These are regression model that are too dependent on the data set at hand, making its value on new data doubtful. The latter strategy, however, refers to computing predictive accuracy of the final model (including health-related quality of life data and known clinical prognostic factors) and comparing it with the predictive accuracy of the model with known clinical prognostic factors only, using for instance, C-indexes [138]. More technical details of Mauer et al. arguments and recommendations can be found elsewhere [139].

This review included all major search engines in combination with a manual search. However, since the strategy was based on keywords in the titles of English language publications, there is a risk that some relevant papers were missed. Furthermore, individual reports were not examined in detail, and so the findings are not all-inclusive. Bottomley and Efficace have also remarked in their editorial comments that it seems necessary to stress that studies on the relationship between quality of life data and survival duration have yielded considerable evidence, but this is still a relatively novel area of research in oncology and has a long way to go. They suggested that more hypothesis-driven prospective studies are needed to provide robust evidence that health-related quality of life data and patient-reported outcomes independently predict survival duration [140].

## Conclusion

The studies reported in this review provide evidence for a positive relationship between quality of life data, or some aspects of quality of life measures, and the duration of survival in cancer patients. Pre-treatment (baseline) quality of life data appeared to provide the most reliable information for helping clinicians to establish prognostic criteria for treating their cancer patients. It is recommended that future studies should use valid instruments, apply sound methodological approaches and adequate multivariate statistical analyses, adjusted for socio-demographic characteristics and known clinical prognostic factors with a satisfactory validation strategy. This strategy is likely to yield more accurate and specific quality of life-related prognostic variables for specific cancers.

## Competing interests

The author declares that they have no competing interests.

## Authors' contributions

The author carried out this review and wrote the manuscript, and prepared all the tables and the figure.

## Supplementary Material

Additional file 1 **Quality of life instruments**. This is an alphabetic list of instruments used in studies of the relationship between quality of life data and survival duration in cancer patients. Click here for file
