# Supplementary material for: Quality of life data as prognostic indicators of survival in cancer patients: an overview of the literature from 1982 to 2008
Source: Health Qual Life Outcomes. 2009 Dec 23;7:102. doi: 10.1186/1477-7525-7-102 (PMC2805623; doi:10.1186/1477-7525-7-102)
Supplement: Additional file 1 — Quality of life instruments. This is an alphabetic list of instruments used in studies of the relationship between quality of life data and survival duration in cancer patients. [file 1477-7525-7-102-S1.DOC]

**The list of instruments used in studies of the relationship between quality of life data and survival duration in cancer patients**

Professor Ali Montazeri

Iranian Institute for Health Sciences Research, ACECR, P.O. Box 13185-1488, Tehran, Iran

E-mail: [montazeri@acecr.ac.ir](mailto:montazeri@acecr.ac.ir)

This is an alphabetic list of instruments used in studies of the relationship between quality of life data and survival duration in cancer patients. The list contains the full name and abbreviations for each instrument.

**The** **list of instruments used in studies of the relationship between quality of life data and survival duration in cancer patients**

**A**

ACS: Adjustment to Cancer Scale;

ADL: Activities of Daily Living;

AQLQ: Auckland Quality of Life Questionnaire;

**B**

BPI: Brief Pain Inventory;

**C**

CECS: Courtauld Emotional Control Scale;

CES-D: Centre for Epidemiologic Studies-Depression Scale;

**D**

DDC: Daily Dairy Card;

**E**

EORTC QLQ-C30: European Organization for Research and Treatment of Cancer Quality of Life Core Questionnaire;

EORTC QLQ-BN20: EORTC Brain Cancer specific Quality of Life Questionnaire;

EORTC QLQ-H&N35: EORTC Head and Neck Cancer specific Quality of Life Questionnaire;

EORTC QLQ-LC13 (or QLQ LC17): EORTC Lung Cancer specific Quality of Life Questionnaire (previously containing 17items);

EORTC QLQ-MY24: EORTC Myeloma specific Quality of Life Questionnaire;

EORTC QLQ-OES18 (previously QLQ-OES24): EORTC Esophageal Cancer specific Quality of Life Questionnaire;

EORTC QLQ-PR25: EORTC Prostate Cancer specific Quality of Life Questionnaire;

ESAS: Edmonton Symptom Assessment System;

ESS: Epworth Sleepiness Scale;

**F**

FAACT: Functional Assessment of Anorexia/Cachexia Therapy;

FACT/GOG-Ntx: Functional Assessment of Cancer Therapy Gynecologic Oncology Group Neurotoxicity scale;

FACT-Br: Functional Assessment of Cancer Therapy-Brain module;

FACT-F: Functional Assessment of Cancer Therapy-Fatigue scale;

FACT-G: Functional Assessment of Cancer Therapy-General module;

FACT-H&N: Functional Assessment of Cancer Therapy-Head & Neck module

FACT-L: Functional Assessment of Cancer Therapy-Lung module;

FACT-P: Functional Assessment of Cancer Therapy- prostate module;

Ferrans and Powers QLI: Ferrans and Power Quality of Life Index;

FLI-C: Functional Living Index-Cancer;

**G**

GHQ: General Health Questionnaire;

**H**

HADS: Hospital and Anxiety Depression Scale;

HDS: Hamilton Depression Scale;

HNQOL: Head and Neck Quality of Life Questionnaire;

**I**

IADL: Instrumental Activities of Daily Living;

IES: Impact of Events Scale;

**K**

KPS: Karnofsky Performance Status;

**L**

LASA: Linear Analog Self-Assessment;

LCSS: Lung Cancer Symptoms Scale;

LSS: Life Satisfaction Score;

**M**

MAC: Mental Adjustment to Cancer Scale;

McGill QOL: McGill quality of Life-single item;

MMSE: Folstein Mini-Mental State Examination;

MOS Social Support Questionnaire: Medical Outcomes Study Social Support Questionnaire

MPAC: Memorial Pain Assessment Card;

MSAS: Memorial Symptom Assessment Scale;

MSAS-GDI: Memorial Symptom Assessment Scale-Global Distress Index;

**N**

NHP: Nottingham Health Profile;

**P**

PAIS: Psychological Adjustment to Illness Scale;

POMS: Profile of Mood States;

POMS-SF: Profile of Mood State-Short Form;

PROSQOL: Prostate Cancer-Specific Quality-of-Life Instrument;

**Q**

QOL-ACD: Quality of Life Questionnaire for Cancer Patients Treated with Anticancer Drugs;

**R**

RMHI: Rand Mental Health Inventory;

RSCL: Rotterdam Symptom Checklist;

**S**

SCL-90-R: Symptom Check List-90 items-Revised;

SDS: Self-rating Depression Scale;

SDS: Symptom Distress Scale;

SF-36: 36-item Short Form Health Survey;

SIP: Sickness Impact Profile;

Spitzer QLI: Spitzer Quality of Life Index;

**T**

TIQ: Therapy Impact Questionnaire;

TOI: Trial Outcome Index;
